# Comparative microelectrode array data of the functional development of hPSC-derived and rat neuronal networks

**DOI:** 10.1038/s41597-022-01242-4

**Published:** 2022-03-30

**Authors:** Fikret Emre Kapucu, Andrey Vinogradov, Tanja Hyvärinen, Laura Ylä-Outinen, Susanna Narkilahti

**Affiliations:** Faculty of Medicine and Health Technology, Tampere University, Tampere, Finland

**Keywords:** Action potential generation, Extracellular recording, Neural stem cells, Scientific data

## Abstract

We present a dataset of microelectrode array (MEA) recordings from human pluripotent stem cell (hPSC)-derived and rat embryonic cortical neurons during their *in vitro* maturation. The data were prepared to assess extracellularly recorded spontaneous activity and to compare the functional development of these neuronal networks. In addition to recordings of spontaneous activity, we provide pharmacological responses of hPSC-derived and rat cortical cultures at their mature stage. Together with the recorded electrode raw data, we share the analysis code to form a comprehensive dataset including spike times, spike waveforms, burst activity and network synchronization metrics calculated with two different connectivity estimators. Moreover, we provide the analysis code that produced the key scientific findings published previously with this dataset. This large dataset enables investigation of the functional aspects of maturing cortical neuronal networks and provides substantial parameters to assess the differences and similarities between hPSC-derived and rat cortical networks *in vitro*. This publicly available dataset will be beneficial, especially for experimental and computational neuroscientists.

## Abstract (summary)

**Table:** 

Measurement(s)	extracellular electrophysiological activity
Technology Type(s)	microelectrode array
Factor Type(s)	days *in vitro* • dosage
Sample Characteristic - Organism	hPSC-derived neuronal cultures • rat neuronal cultures
Sample Characteristic - Environment	functional development • pharmacological response

## Background & Summary

Human pluripotent stem cell (hPSC)-derived neurons are becoming a more prominent tool for *in vitro* disease modeling, neurotoxicology and drug research with great prospects for reducing animal studies. Such patient-specific models have the potential to unravel the cellular and functional mechanisms behind neurological diseases^1^. Compared to rodent counterparts, generating electrophysiologically mature neuronal networks from hPSCs has been challenging. However, during the last decade, several protocols and methodologies have been introduced to improve differentiation^2,3^, culture conditions^4,5^ and functional maturation^6,7^. With these improvements, hPSC-derived neurons have claimed their place as a valuable *in vitro* tool.

Microelectrode arrays (MEAs) have been used to measure neuronal electrophysiology extracellularly *in vitro*, *in vivo* and *ex vivo*^8–10^. The primary advantage of MEAs over cellular-level electrophysiological measurement methods is their ability to measure the activity of a population of neurons simultaneously to provide information about network behavior, thus allowing analyses of network-wide properties, e.g., connectivity^11^. In addition, the noninvasive nature of the technique enables repeated measurements over time, allowing follow-up on developmental events and prolonged responses to different exposures, e.g., pharmacological responses^12^. Importantly, commercially available multiwell MEAs increase throughput in measurements^6,13^. Typically, network activity starts with individual spiking, followed by spike trains and further mature burst behavior^8,14^. These developmental steps and their features can be analyzed in depth to understand the activity pattern development of networks. As rodent neurons have been the gold standard in the field, MEA culture techniques, specific studies and signal analysis protocols have been established primarily with them^14,15^. Today, more data also exist for hPSC-derived neuronal networks^12,16–18^.

**Fig. 1:**
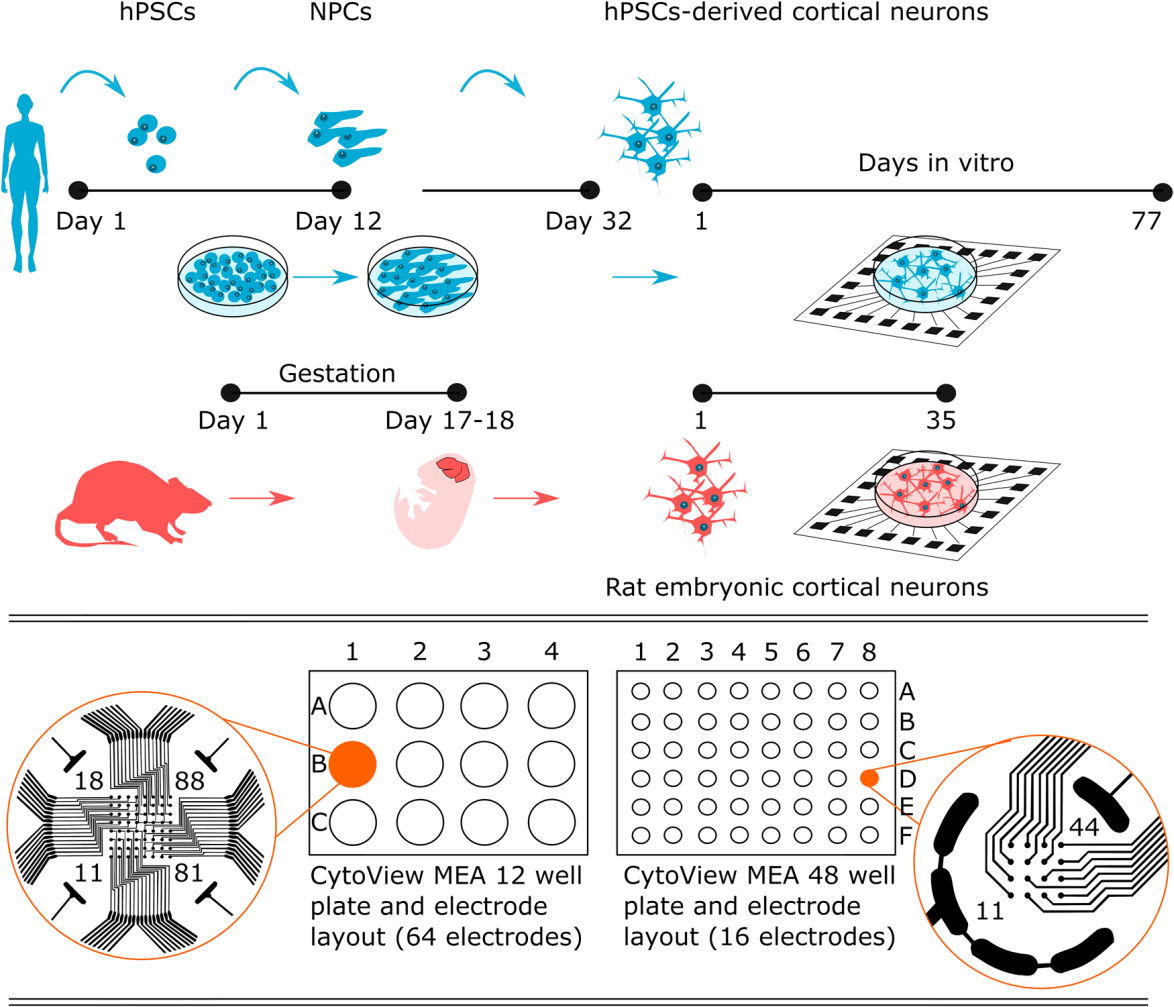
Experimental setup for following the functional development of human and rat networks on two different MEA well plate types. After human pluripotent stem cells (hPSCs) differentiated into cortical neurons, they were plated on CytoView MEA 12 and CytoView MEA 48 well plates. Rat embryonic cortical cells were plated on the same MEA well plate types in parallel. Regular timepoint recordings were obtained in CytoView MEA 12, which has 64 electrodes per well, and pharmacological experiments were performed on CytoView MEA 48, which has 16 electrodes per well. Measurement days in MEA are referred to as days *in vitro* (DIV). Pharma recordings were obtained at DIV 29 and DIV 22, when peak activity is commonly observed for *in vitro* human and rat neuronal networks, respectively.

Previously, we used MEA technology to characterize the development of hPSC-derived neuronal network activity and compared it with rat embryonic cortical networks^17^ (Fig. 1). We followed the spontaneous activity of the developing networks with regular time-point recordings and recorded network responses to pharmacological manipulations at a single time point. The pharmacology recordings were collected at a single time point to assess spontaneous baseline recordings and subsequent electrophysiological responses to glutamatergic and GABAergic agonists and antagonists as well as voltage-gated sodium channel blockers.

From the data point of view, to our knowledge, there are no openly available MEA datasets covering both hPSC-derived and rodent neuronal networks acquired in similarly designed and parallel experiments. Here, we share an approximately 2 TB dataset covering approximately 740 minutes of raw MEA recordings. The provided dataset can be compared by means of activity development patterns; thus, it can be used to build a comparative model for cortical activity development and network formation in such cultures. The recordings can be further analyzed with different analysis methods than were previously used by us. It is common for different labs to use their own analysis methods for data evaluation. For these reasons, we provide not only the raw data but also the outputs of the analyses that we previously performed. Thus, we provide a full dataset including spike times and necessary analysis code such as burst analysis code to detect burst start and end times and subsequently calculate burst statistics. Principal component analysis (PCA)-based classification results and network synchronization values calculated with two different connectivity estimators were also included in the dataset. Users can also harvest other types of parameters or different types of data, such as spike waveforms, from the raw data with small additions to the code we provide.

This pharmacological dataset may be especially beneficial in testing changes in network connectivity since the data were collected from functionally mature networks. As spontaneous activity, the response to pharmacological treatments can be analyzed by means of interdependences between spike time series, thus revealing network formation^19^ and information transfer between neurons^20,21^.

Overall, our shared datasets provide an opportunity for MEA users in the neuroscience community to evaluate their own data quality and analysis tools to assess “**developing neuronal activity** ***in vitro***”. Moreover, the data can also be used as validation for in silico neuronal network models and simulations^22^. In conclusion, revealing the original data provides an opportunity to the experimental and computational neuroscience community for various purposes of reuse and thorough analysis between different types of networks.

## Methods

Methods explained below are expanded versions of descriptions in our related work^17^.

### hPSCS lines and neural differentiation

The hPSC lines utilized in this study, Regea 08/023 and 10212.EURCCs, have been previously characterized^23,24^. The study was approved by the regional ethics committee of Pirkanmaa Hospital District (R05116 and R08070). hPSCs were cultured in feeder-free conditions on a defined substrate, laminin 521 (LN521, Biolamina, Sweden), according to the protocol of Hongisto and colleagues^25^. The detailed neural differentiation protocol is described in Hyvärinen *et al*. 2019^17^. The MEAs were coated with 0.1% polyethyleneimide (PEI, Sigma, CAS:9002–98–6) and 50 µg/ml LN521. At day 32, the cells were plated for MEAs at a density of 1 × 10^6^ cells/cm^2^. Neural maintenance medium was used when cells were cultured on MEAs. The medium consisted of 1:1 DMEM/F12 with GlutaMAX and Neurobasal, 0.5% N2, 1% B27 with retinoic acid, 0.5 mM GlutaMAX, 0.5% NEEA, 50 µM 2-mercaptoethanol (all obtained from Thermo Fisher Scientific, CAS:60–24–2), 2.5 µg/ml insulin (Sigma, CAS:11061–68–0) and 0.1% penicillin/streptomycin (Thermo Fisher Scientific). Neural maintenance medium was supplemented with 20 ng/ml brain-derived neurotrophic factor (BDNF, R&D Systems), 10 ng/ml glial-derived neurotrophic factor (GDNF, R&D Systems), 500 µM dibutyryl-cyclicAMP (db-cAMP, Sigma, CAS:16980–89–5) and 200 µM ascorbic acid (AA, Sigma, CAS:50–81–7). Medium was changed every two to three days.

### Primary rat cultures

Primary cells were obtained from the Neuronal Cell Culture Unit at the University of Helsinki, Finland. Cortex tissue was harvested from Wistar rat embryos (E17-18) as described previously^26^. The described procedures were conducted under the animal license (County Administrative Board of Southern Finland, ESAVI/10300/04.10.07/2016) and approved by local authorities. All described experiments were performed in accordance with institutional guidelines and regulations (University of Helsinki internal license number: KEK17–016). MEA plates were coated with 25 µg/ml poly-D-lysine (PDL, Sigma, CAS:27964-99-4). The cells were plated at a density of 2.5 × 10^5^ cells/cm^2^ for MEAs. Cortical neurons were cultured in medium consisting of Neurobasal, 2% B27, 2 mM GlutaMAX and 1% penicillin/streptomycin (Thermo Fisher Scientific). Medium was changed every two to three days.

### Data acquisition tools

Extracellular recordings were acquired with an Axion Maestro system controlled by AxIS software version 2.4 (Axion Biosystems, Atlanta, GA, USA) with a sampling rate of 12.5 kHz as previously described^6^. The recording hardware characteristics were configured to 1200 × gain and a bandwidth of 10–5000 Hz. Cells were plated on *CytoView MEA 12* M768-GL1-30Pt200 for recording spontaneous activity development and on *CytoView MEA 48* M768-tMEA-48W for pharmacological experiments (both from Axion Biosystems). The *CytoView MEA 12* plates contained 64 recording electrodes per well, and the *CytoView MEA 48* plates contained 16 recording electrodes per well. The electrode layouts are presented in Fig. 1.

### Microelectrode array measurements

Recordings were performed at 37 °C, and a 5% CO2 atmosphere was secured during measurements exceeding 10 minutes. Spontaneous activity was recorded twice a week for 10 minutes. We collected recordings from DIV 3 to DIV 77 for hPSC-derived cortical neurons and DIV 2 to DIV 35 for rat cortical neurons, which covered all the analyses performed previously^17^ (Table 1).

**Table 1:** Information on the shared MEA data from each MEA well plate grouped according to different types of assessments in previous work^17^.

MEA well plate name	Cell type	Wells used/Well plate	Time points (days *in vitro*)	Additional Information	Located in Folder
hPSC_MEA1	hPSC	6/12	3–66	Regular timepoint recording	**hPSC_MEA1**
hPSC_MEA2	hPSC	6/12	3–66	Regular timepoint recording	**hPSC_MEA2**
hPSC_MEA1	hPSC	6/12	21, 24, 28	Regular timepoint recording	**PCA**
hPSC_MEA2	hPSC	6/12	21, 24, 28	Regular timepoint recording	**PCA**
hPSC_MEA3	hPSC	24/48	29	Pharma experiments	**hPSC_MEA3_ Pharmacology**
hPSC_MEA4	hPSC	6/12	21, 24, 28	Used for data classification with PCA	**PCA**
hPSC_MEA5	hPSC	4/12	70, 73, 77	Used for data classification with PCA	**PCA**
Rat_MEA1	Rat	12/12	2–35	Regular timepoint- recording	**Rat_MEA1**
Rat_MEA1	Rat	12/12	21, 24, 28	Regular timepoint- recording	**PCA**
Rat_MEA2	Rat	42/48	22	Pharma experiments	**Rat_MEA2_ Pharmacology**
Rat_MEA3	Rat	12/12	21, 24, 28	Used for data classification with PCA	**PCA**
Rat_MEA4	Rat	12/12	24, 28, 31	Used for data classification with PCA	**PCA**

The recording time points are also given to clarify the purpose of having copies of the same recordings in different folders, as some of the recordings were used in more than one assessment.

Pharmacological experiments were performed at the time point when peak activity was commonly observed; for hPSC-derived networks, this was at DIV 29, and for rat cortical networks, it was at DIV 22 (Table 1). Both baseline activity and the subsequent pharmacological responses were recorded for 30 minutes. The following reagents were used: kainic acid (KA, 5 µM, Sigma, CAS:58002-62-3), α-amino-3-hydroxy-5-methyl-4-isoxazolepropionic acid (AMPA)/kainate receptor antagonist 6 cyano 7 nitroquinoxaline 2,3 dione (CNQX, 50 µM, Abcam, CAS:115066-14-3), N-methyl-D-aspartate (NMDA) receptor antagonist D-(-)-2 amino-5-phosphonopentanoic acid (D-AP5, 50 µM, Sigma, CAS:79055-68-8), γ-aminobutyric acid (GABA, 10 µM, Sigma, CAS:56-12-2), and GABAA receptor antagonist gabazine (30 µM, Sigma, CAS:104104-50-9). Pharmacological experiments were followed by the application of the voltage-gated sodium channel blocker tetrodotoxin (TTX, 1 µM, Tocris, CAS:18660-81-6). The response to TTX was recorded for 10 minutes. The final concentrations mentioned above were achieved by adding 30 µl of pharmacological agents at higher concentrations into the MEA wells.

### MEA datasets

The datasets in this article are described under three different categories based on the purpose of collection: regular timepoint recordings, recordings of pharmacological experiments and recordings selected for PCA-based classification analysis (Table 1). These categories are briefly described as follows:

- The dataset of regular timepoint recordings contains recordings of spontaneous activity performed with *CytoView MEA 12*-well plates (Fig. 1). For human cells, recordings were obtained from MEA well plates hPSC_MEA1 and hPSC_MEA2 from DIV 3 to DIV 66. Recordings from rat cells were obtained from MEA well plate Rat_MEA1 from DIV 2 to DIV 35. Cells that were plated on hPSC_MEA1 and hPSC_MEA2 were from the same differentiation batch and were recorded in parallel; thus, the recorded MEA data can be pooled as in Hyvärinen *et al*.^17^.
- The Pharma dataset contains recordings during pharmacological experiments. All recordings were performed with *CytoView MEA 48*-well plates (Fig. 1). The MEA well plate hPSC_MEA3 was recorded at DIV 29, and Rat_MEA2 was recorded at DIV 22, when peak activity is commonly observed for *in vitro* human and rat neuronal networks, respectively.
- The dataset that was formed for PCA-based classification includes recordings from a dataset of regular timepoint recordings (hPSC_MEA1, hPSC_MEA2 and Rat_MEA1). Two additional MEA well plates were included in the dataset for both human (hPSC_MEA4 and hPSC_MEA5) and rat (Rat_MEA3 and Rat_MEA4) data groups. All included recordings were performed with *CytoView MEA 12*-well plates (Fig. 1). The human MEA data were derived from two neural differentiations of human embryonic stem cells (hESC) line 08/023 (hPSC_MEA1, hPSC_MEA2 and hPSC_MEA4) and one of human induced pluripotent stem cells (hiPSC) line 10212.EURCCs (hPSC_MEA5). The rat MEA data were derived from three independent batches of rat embryonic neurons (Rat_MEA1, Rat_MEA3 and Rat_MEA4). From each MEA dataset, the time point showing the most significant activity (i.e., the maximum spike rate) and two of its surrounding time points were selected for PCA. The selected time points were DIV 21, 24 and 28 for hPSC_MEA1, hPSC_MEA2 and hPSC_MEA4; DIV 70, 73 and 77 for hPSC_MEA5; DIV 21, 24 and 28 for Rat_MEA1 and Rat_MEA3; and DIV 24, 28 and 31 for Rat_MEA4 (Table 1).

### Data preparation

The measurements were recorded as .raw files by acquisition software, which contained voltage values as a product of a voltage scale factor and a set of integer values stored separately during signal quantization. The published data were reformatted to HDF5 (.h5) file format with a custom MATLAB script; thus, the reformatted raw recordings have only the final calculated voltage values for ease of use.

**Fig. 2:**
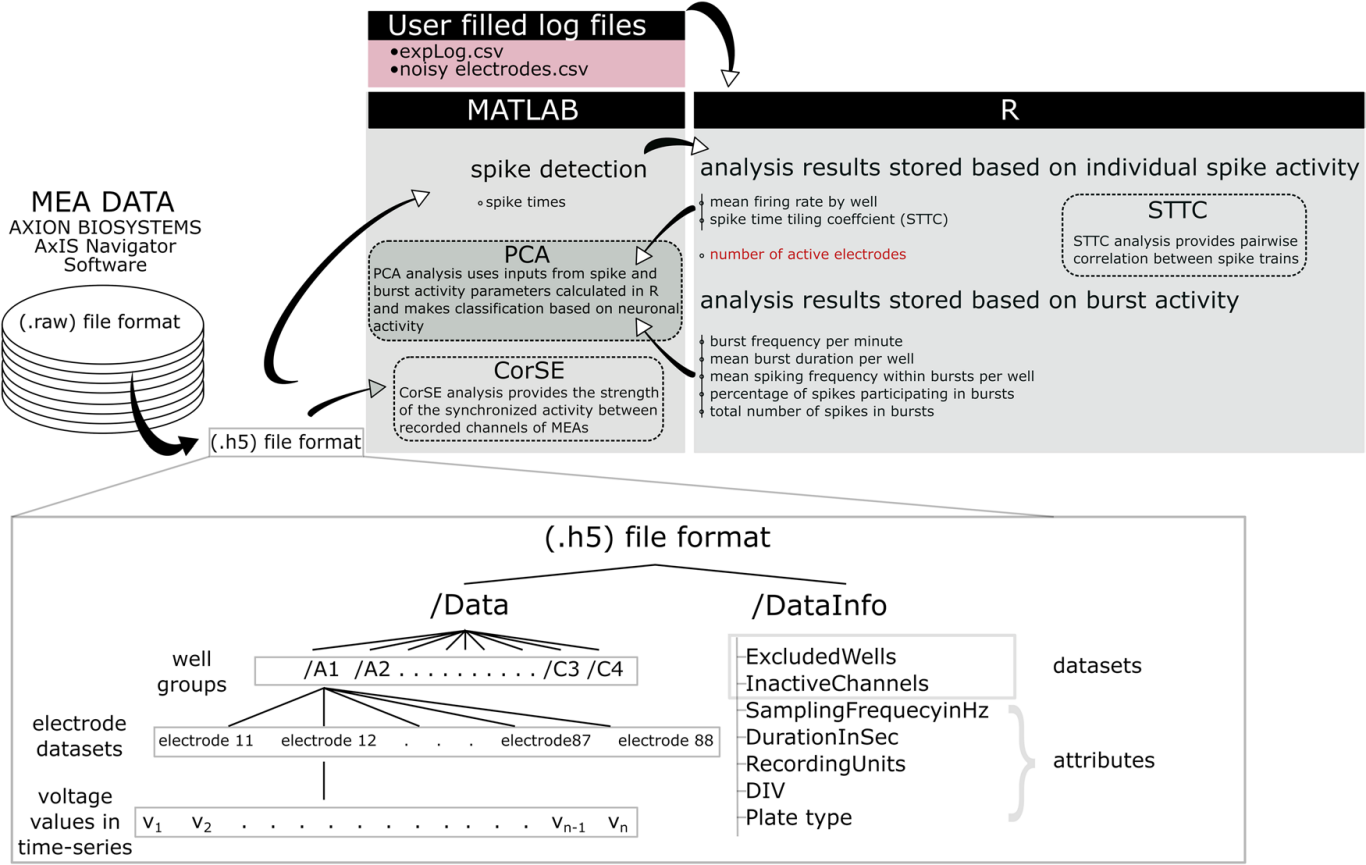
Preparation and analysis of the data. The raw data recorded with AxIS Navigator software were reformatted in HDF5 format (.h5) to include both the raw data and associated metadata. The architecture of .h5 is also presented. Spike detection was performed in MATLAB, and the spike times were saved for further analysis. Burst detection was performed in R from the previously calculated spike times. The parameters based on spike and burst activity were calculated in R, and the results were stored in .csv format. The stored parameters were used as inputs to principal component analysis (PCA) for data classification (except the number of active electrodes, which was only used for calculating some other parameters). Network synchronization was estimated from the raw data by correlated spectral entropy (CorSE) analysis in MATLAB, and the spike time tiling coefficient (STTC)-based pairwise correlation was calculated from the spike times in R.

We also stored metadata in each HDF5 file. Thus, each HDF5 file contains two main groups: ‘/Data’ and ‘/DataInfo’. Hierarchically, the ‘/Data’ group contains groups for each well, each well group contains a number of datasets equal to the number of electrodes, and each dataset contains a time series of the voltage values (Fig. 2). The ‘/DataInfo’ group contains two datasets and five attributes, including the information on the excluded wells, inactive channels for each recorded well, sampling frequency, duration of the recording, recording units (i.e., volts), time point of the measurement and MEA well plate type used.

### Microelectrode array data analysis

Analysis of the MEA data consisted of several consecutive steps starting with the assessment of the raw data. In the initial stage of the analysis, spikes were extracted from the .h5 formatted data, with spike detection performed in MATLAB. The detected spike times were stored with their corresponding electrode labels in .csv files. Analyses based on individual spike activity and bursts were performed in R with a modified version of meaRtools^27^. Bursts were detected, and their start and end times were labeled. After labeling the bursts, several parameters associated with bursting were calculated and stored in .csv files with corresponding names. For synchronized activity, the spike time tiling coefficients (STTC) were also calculated in R^27^ and stored in .csv files. PCA was performed in MATLAB according to the parameters associated with the spike firing statistics, burst parameters and synchronization analysis, i.e., STTC. In addition, we analyzed network connectivity from the raw data based on the correlations of the time-variant spectral entropies (CorSE)^19^. Fig. 2 presents the overall analysis process used in this work. The required code to reproduce the shared results is also provided with this article.

#### Spike detection

Spike detection was performed according to the stationary wavelet transform-based Teager energy operator (SWTTEO) algorithm presented previously^28^ and revised for biological data^29^. The algorithm was embedded into custom MATLAB (MathWorks) code. During initial tests, the performance of the method in detecting the low-amplitude spikes typical for hPSC-derived networks was confirmed^17^. In short, the data were prefiltered with an elliptic bandpass filter with a passband range of 200–3000 Hz. First, initial absolute-amplitude-based thresholding^30^ was performed with the threshold value, which was set to 4.5 times the estimate of the standard deviation of the noise. During the next step, putative false positive spikes were cancelled using the SWTTEO algorithm. Briefly, the total number of spikes extracted by threshold detection was used by SWTTEO analysis to detect the same number of spikes, and only the spikes detected by both methods were considered true positives. Electrodes exhibiting >10 spikes per minute were considered active and included in further analysis.

#### Burst detection and analysis

We used the logISI algorithm for burst detection^31^ with small modifications. The minimum number of spikes in a burst was set to 5. Another adjustment was applied to merge bursts if their interburst interval was less than 100 ms. Then, the bursts were analyzed to calculate the associated parameters. Burst analysis was performed with the R package meaRtools^27^ only for the electrodes exhibiting bursts. Notably, this burst detection algorithm was not introduced in the original meaRtools, but we integrated it into the analysis code shared with this article.

#### Connectivity analysis

Network connectivity was analyzed using the CorSE method described earlier^19^. Functional connectivity was assessed pairwise in each MEA well. In summary, CorSE evaluates the synchronization of signals by the correlation of the temporal changes in their spectral contents. The magnitude of this correlation represents the connectivity strength. The average CorSE values were calculated from all channels of the MEA well to assess the overall connectivity strength of the whole network. To observe the changes in network formation, connectivity maps were plotted for the channel pairs of the most robust network participants, e.g., for the channels with CorSE > 0.7, as in Hyvärinen *et al*.^17^.

#### Principal component analysis

PCA was used as previously described^32^ to observe the difference in network activity in different experiments. A list of 7 features from spike, burst and network synchronization analysis were selected for comparison. These included the *mean firing rate by active electrodes (MFR), burst rate (per minute), mean burst duration (in seconds), mean spike frequency in bursts (spikes/burst duration), mean number of spikes in a burst, percentage of spikes in bursts and spike time tiling coefficient (STTC, using a default time bin of 50 msec)*. All values were normalized with the standard score approach before the analysis. PCA was implemented in MATLAB. The three major principal components were selected, and a three-dimensional plot was generated.

#### Statistical analysis

We do not share any produced results from statistical analysis in this work. However, statistical analysis can be performed on the output of the meaRtools analysis which we also shared. In the original article^17^ nonparametric Mann-Whitney U test was used for statistical analysis (p-value < 0.05 was considered significant). Tests were performed with SPSS Statistics software (version 25.0).

## Data Records

The data shared in this paper were prepared and processed in several consecutive processes (Fig. 2). From the acquisition of the raw data to the end stage analysis, we primarily analyzed the data in MATLAB and R. The analysis output was stored in .csv file format (Fig. 2). The post analysis and figures were produced from these.csv files. The file names were created by using the labels of the MEA well plate used, the day of the culturing and the time point of the recording. For example, the <***data label****>* for the MEA well plate hPSC_MEA1, which was plated on 02/05/17 (dd/mm/yy) and recorded on DIV 35, is *hPSC_20517_MEA1_DIV35*. For the pharmacological experiments, the MEA plate label continues with a specific postfix such as “Baseline”, “Pharma” or “TTX”, which indicates the corresponding stage of the experiment. The data in the repository are separated into folders, where each folder is associated with a single MEA well plate. The folders can be found in https://gin.g-node.org/NeuroGroup_TUNI/Comparative_MEA_dataset^33^. Each of these folders contains raw data files in *.h5* format, the previously calculated spike and burst parameter results in *.csv* format, a noisy electrode list in .csv format and the expLog files used by the analysis code in *.csv* format.

Briefly, the files in these folders can be described as follows:

- **<data label>.h5 files:** the raw recording from each MEA well plate with associated metadata. Architecturally, each HDF5 file contains *‘/Data*’ and ‘/*DataInfo*’ groups.
  - The ‘/Data’ group includes subgroups for each well of the MEA plate, e.g., ‘/Data/A3’. Each of these subgroups includes a number of datasets equal to the number of electrodes. Each dataset is named for the corresponding electrode and contains a time series of voltage values.
  - The ‘/DataInfo’ group contains the information on the data recorded: two datasets, ‘ExcludedWells’ and ‘InactiveChannels’, and five attributes, ‘SamplingFrequencyInHz’, ‘DurationInSec’, ‘RecordingUnits’, ‘DIV’, and ‘Plate type’.
    - *ExcludedWells*: the list of excluded wells of the MEA plate that were not used for the experiment
    - *InactiveChannels*: the list of single channels in the MEA plate that were not recorded due to malfunctioning electrodes during acquisition
    - *SamplingFrequencyinHz*: sampling frequency of the recording
    - *DurationInSec*: duration of the recording in seconds
    - *RecordingUnits*: Volts
    - *DIV:* days *in vitro* on MEA on the day of recording
    - *Plate type*: MEA plate type, either 12 or 48 wells per plate
- **noisy_electrodes_<MEA well plate name>.csv files:** the list of noisy electrodes for meaRtools, which are automatically discarded by the analysis code.
- <**data label>_expLog.csv files:** the log files for meaRtools, which are named in accordance with the first DIV included in each set of recordings. They contain the list of wells associated with a particular MEA plate and the corresponding pharmacological treatments, if applied. The data analysis code automatically reads the related information.
- <**data label>_spikes.csv:** spike times calculated in seconds, which are detected from the raw recordings by the analysis code Main.m in MATLAB.

In addition to these files, the output of the meaRtools analysis is returned in the .csv tables. Each table in the .csv files contains a particular output feature value averaged over each well of an MEA plate for each day of recording. A detailed description of the meaRtools package can be obtained from the original publication^27^. The list of output features used in the original work is introduced below:

- **< data label** **>_meanfiringrate_by_active_electrodes.csv:** mean firing rate of the active electrodes in a well
- **< data label >_nae.csv:** number of active electrodes
- **< data label >_STTC.csv:** spike train tiling coefficient evaluating correlations between pairs of electrodes
- **< data label >_bursts_per_min.csv:** average number of bursts per minute
- **< data label >_mean_dur.csv:** average burst duration in seconds for the bursts captured
- **< data label >_mean_freq_in_burst.csv:** average spike frequency for the bursts captured
- **< data label >_per_spikes_in_burst.csv:** percentage of spikes participating in bursts
- **< data label >_mean_spikes_in_burst.csv:** average number of spikes in bursts

In this section, to enhance readability, we present a short summary of each folder’s content in Table 1. For more thorough reading, a description of each file and their format, size and repository address are presented in Supplementary Table 1.

## Technical Validation

**Fig. 3:**
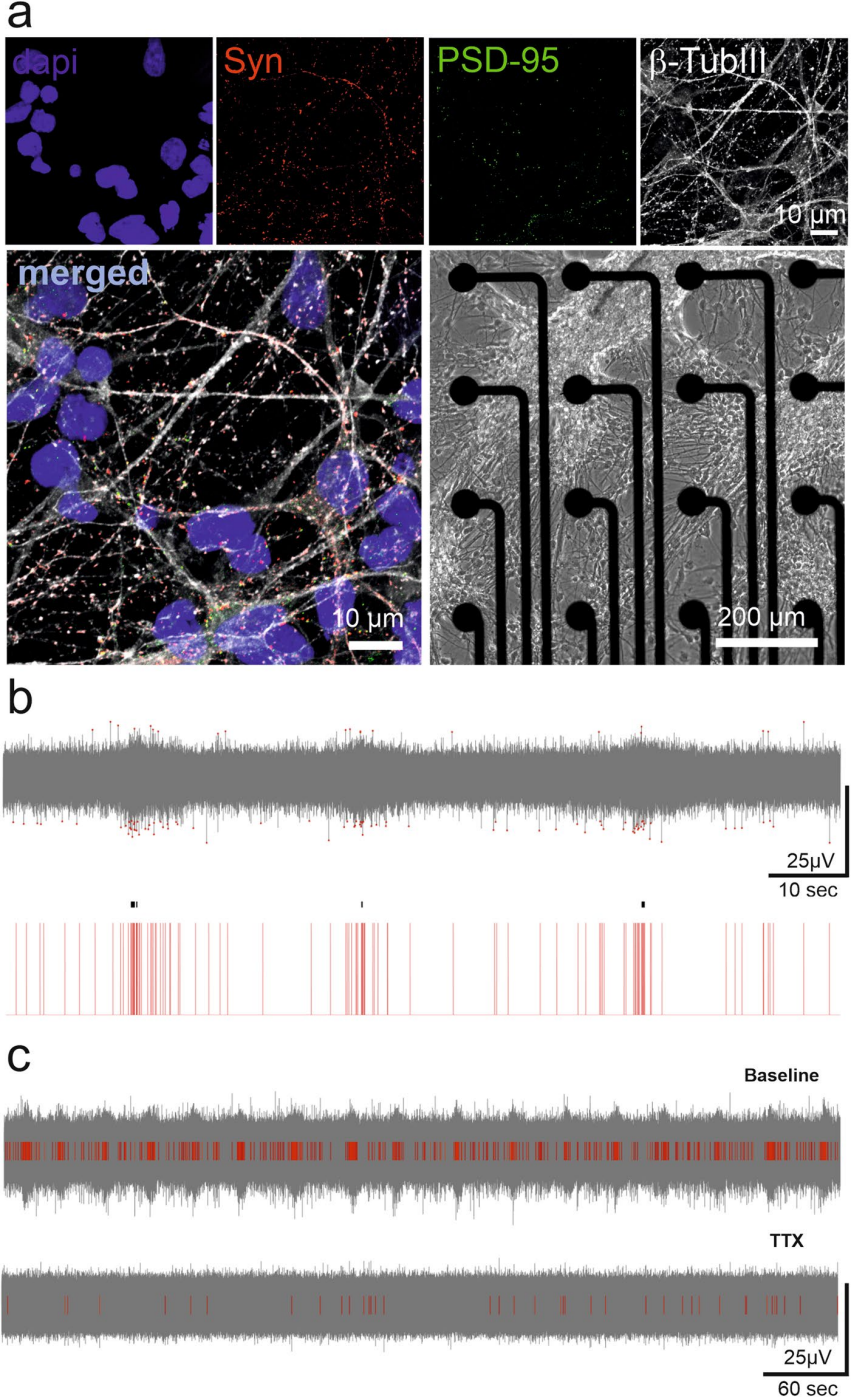
Validation of the MEA recordings of neuronal networks and signal analysis. (**a**) The quality of hPSC-derived neural cultures was verified with immunocytochemical staining of pre- and postsynaptic markers (synaptophysin in red and PSD-95 in green) overlaid with neuron-specific b-tubulin staining (white). DAPI stains cell nuclei. A phase contrast image of hPSC-derived neurons on the MEA is used to illustrate the formation of networks over the electrode area. (**b**) The performance of spike detection in recognizing the typical low-amplitude signal of hPSC-derived networks was confirmed. The detected spikes are illustrated with red dots (top) and red bars (bottom). A single channel burst detection algorithm successfully detected quasiperiodic burst activity, which is commonly observed in mature neuronal cultures. The detected bursts are labeled with black lines on the red spike bars (bottom). (**c**) The MEA signal was associated with action potentials, as it was blocked by the voltage-gated sodium channel antagonist tetrodotoxin (TTX). The number of detected spikes (red bars) decreased considerably (bottom) compared to the baseline recording (top).

The technical quality of MEA recordings depends on several parameters, such as electrode noise, signal-to-noise ratio, amplification, filtering, etc. The parameters directly related to the quality standards of data acquisition are mainly validated by the manufacturer and are distributed upon request (https://www.axionbiosystems.com/). Here, we validated the raw MEA data, whether from a biological source or noise, by silencing the activity with the voltage-dependent sodium channel blocker tetrodotoxin (TTX, Fig. 3c), which blocks neuron-specific signaling. As a result, we confirmed that the activity recorded with the MEAs originated from neuronal activity. Recording channels containing severe artifacts were discarded from analysis. We share a discarded noisy electrode list in .csv files for each MEA. The quality of our cultures was confirmed with parallel assays on cells cultured on glass cover slips (Fig. 3a)^17^. The quality of the analysis tools was also considered; e.g., the spike and burst analysis tools were previously published and are openly available in their originally published forms^28,30,31^; their compatibility with our data was evaluated qualitatively by experts in our group, and necessary modifications were applied. Fig. 3b illustrates an exemplary burst and spike detection result on a sample recording. In this article, we share the analysis code used together with our data.

## Usage Notes

The shared data can be read and assessed with the analysis code provided here or with any other tools that users choose. To access and manipulate the data beyond the provided code, the user needs an appropriate platform supporting the HDF5 file type, for example, MATLAB, Python, R, or Java.

In general, the code is modified to be user-friendly; e.g., the file selection process is organized with the help of pop-up windows with labels that describe the corresponding selection step. However, minor code modifications are still required for some specific cases. Thus, the user should read the comments in the code, which requires basic knowledge of R and MATLAB operations.

The analysis code embeds the necessary commands for reading HDF5 files. To access a particular dataset outside of our code and to obtain raw data, the user needs to use specific commands for HDF5 file reading with the corresponding HDF5 file name and the desired dataset address as its parameters. For example, to access the dataset containing the signal of electrode 22 of well A3 recorded in the ‘hPSC_20517_MEA2_DIV35.h5’ file, the user needs to implement the following MATLAB command:

- *h5read(‘…path/hPSC_20517_MEA2_DIV35.h5’, ‘/Data/A3/22’)*

The same is true of the attributes of the ‘/DataInfo’ group; for example, the duration of the recording is accessible as:

- *h5readatt(‘…path/hPSC_20517_MEA2_DIV35.h5’, ‘/DataInfo’, ‘DurationInSec’)*

(note that the attribute name is specified as a separate argument here).

***For the second argument of the reading functions, which specifies the address inside the .h5 file, the usage of the slash and not the backslash is important*** *.*

An attempt to read datasets for electrodes of wells that are not recorded will lead to an error. These wells are listed in the ‘ExcludedWells’ dataset in the ‘\DataInfo\’ group. The same is true for inactive electrodes stored in the ‘InactiveChannels’ dataset of the same group.

To launch the MATLAB spike detection code, the user needs to open the *Main.m* file and then add the whole folder containing the MATLAB analysis code to the MATLAB path (so that the program is capable of finding the functions that the code requires). Next, by pressing MATLAB’s green “Run” button, the analysis is launched. The selection of the .h5 raw data files for the analysis is implemented via a pop-up window that opens as the user launches the code. One or more files can be selected for analysis. The output folder selection is performed in the same way. The code sequentially analyses the selected files, providing the user with spike .csv files as an output. If the user decides to use different parameters than described in the methods section for spike detection, the parameters to be changed are located in *Main.m* and *amp_detect.m*.

To implement the analyses in meaRtools, the user needs to follow the steps specified below:

i. First, the user needs to open the *MEA_analysis_Axion.R* file.
ii. Then, by clicking the “Source” button, the code is launched. The user sees pop-up windows for selecting the code-containing folder, the output folder, the spike.csv files, the noisy electrode file and the expLog file.
iii. The last pop-up window enables the selection of the analyzed MEA type. There are 12- and 48-well MEA plates available, and the user only needs to enter an integer that corresponds to the analyzed plate type.

***The folder selection windows sometimes do not appear on top of the RStudio window; then, they are found in the Windows taskbar. If the user wants to avoid the code directory selection step, it is possible to remove the first pop-up window by replacing the first “choose.dir” function with the code directory address*** *.*

It should be noted that electrodes that

- have no detected spikes (are not mentioned in the MATLAB-generated .csv spike files)
- are listed in the noisy electrode .csv file
- are eliminated by the minimum-spikes-per-minute criterion

are not included in the meaRtools analysis.

***Essentially, if for these abovementioned reasons all electrodes in a particular well are cancelled for all DIVs included in the analysis, this well is not displayed in the analysis output files*** *.*

There is a possibility of implementing the code in segments by performing segment selection and pressing the “Run” button.

The PCA and connectivity analysis MATLAB code packages are delivered in their corresponding folders. To run the PCA code, the user needs to open the *PCA.m* code in the “PCA” folder and add the whole folder to the MATLAB path. The folder contains a table with preselected activity features of the cell populations obtained during the provided analysis path. The next step is to click the “Run” button to launch PCA.

To launch the connectivity analysis, the user needs to open the *Connectivity.m* file in the “Connectivity analysis” folder and add this folder to the MATLAB path. After clicking the “Run” button, the pop-up window for .h5 file selection appears. After selecting the desired file, a new pop-up window for MEA well selection appears. The user needs to take into account the list of excluded wells, which are automatically displayed in the command window after file selection, and avoid selecting them. For the connectivity analysis, the threshold value for connectivity strength can be changed in the script *cross_selection_correlated_channels.m*. More information on the CorSE and analysis guidelines can be found at https://se.mathworks.com/matlabcentral/fileexchange/59626-spectral-entropy-based-neuronal-network-synchronization-analysis-corse.

## Supplementary information

Supplementary Table 1

## Data Availability

The provided codes are modified versions of those published earlier^19,27,28,31^ and custom in-house scripts. Modification and further distribution fall under the restrictions described by the authors. The codes can be found in https://gin.g-node.org/NeuroGroup_TUNI/Comparative_MEA_dataset/src/master/Codes^33^. MATLAB 2020a (MathWorks) and RStudio version 1.3.959 were used during the preparation of the current publication.
